# Whole-genome DNA/RNA sequencing identifies truncating mutations in *RBCK1 *in a novel Mendelian disease with neuromuscular and cardiac involvement

**DOI:** 10.1186/gm471

**Published:** 2013-07-26

**Authors:** Kai Wang, Cecilia Kim, Jonathan Bradfield, Yunfei Guo, Elina Toskala, Frederick G Otieno, Cuiping Hou, Kelly Thomas, Christopher Cardinale, Gholson J Lyon, Ryan Golhar, Hakon Hakonarson

**Affiliations:** 1 Zilkha Neurogenetic Institute, Keck School of Medicine, University of Southern California, 1501 San Pablo St, Los Angeles, CA 90089, USA; 2 Center for Applied Genomics, Children's Hospital of Philadelphia, 3615 Civic Center Blvd, Philadelphia, PA 19104, USA; 3 Stanley Institute for Cognitive Genomics, Cold Spring Harbor Laboratory, One Bungtown Rd, NY 11724, USA; 4 Department of Pediatrics, University of Pennsylvania School of Medicine, 3451 Walnut St, Philadelphia, PA 19104, USA

## Abstract

**Background:**

Whole-exome sequencing has identified the causes of several Mendelian diseases by analyzing multiple unrelated cases, but it is more challenging to resolve the cause of extremely rare and suspected Mendelian diseases from individual families. We identified a family quartet with two children, both affected with a previously unreported disease, characterized by progressive muscular weakness and cardiomyopathy, with normal intelligence. During the course of the study, we identified one additional unrelated patient with a comparable phenotype.

**Methods:**

We performed whole-genome sequencing (Complete Genomics platform), whole-exome sequencing (Agilent SureSelect exon capture and Illumina Genome Analyzer II platform), SNP genotyping (Illumina HumanHap550 SNP array) and Sanger sequencing on blood samples, as well as RNA-Seq (Illumina HiSeq platform) on transformed lymphoblastoid cell lines.

**Results:**

From whole-genome sequence data, we identified RBCK1, a gene encoding an E3 ubiquitin-protein ligase, as the most likely candidate gene, with two protein-truncating mutations in probands in the first family. However, exome data failed to nominate RBCK1 as a candidate gene, due to poor regional coverage. Sanger sequencing identified a private homozygous splice variant in RBCK1 in the proband in the second family, yet SNP genotyping revealed a 1.2Mb copy-neutral region of homozygosity covering RBCK1. RNA-Seq confirmed aberrant splicing of RBCK1 transcripts, resulting in truncated protein products.

**Conclusions:**

While the exact mechanism by which these mutations cause disease is unknown, our study represents an example of how the combined use of whole-genome DNA and RNA sequencing can identify a disease-predisposing gene for a novel and extremely rare Mendelian disease.

## Background

Over 5,500 confirmed Mendelian diseases have been described in the Online Mendelian Inheritance in Man (OMIM) database as of June 2013, but a third of them do not have a known molecular basis. With the rapid development and deployment of next-generation sequencing techniques, this situation is changing rapidly [1,2]. Over the past a few years, exome sequencing has been successfully used to identify candidate predisposing genes for multiple Mendelian diseases, and it is likely that this technique will impact clinical medicine in the relatively near future [3,4]. However, we also note two important points from recently published studies. First, the vast majority of Mendelian sequencing studies used exome sequencing rather than whole-genome sequencing. This is due to several reasons, such as the lower cost of exome sequencing, the assumption that Mendelian diseases are more likely to be caused by mutations at exons than non-coding regions, and the concern that too much information on genomic variants will be too difficult to interpret bioinformatically. Second, the vast majority of published studies attempted to solve previously known Mendelian diseases, rather than novel suspected Mendelian phenotypes that are sometimes referred to as 'idiopathic' diseases. This is most likely because multiple DNA samples are already readily available for known Mendelian diseases to enable statistical support for discovered variants/genes. However, several examples demonstrated that it is feasible to identify disease-predisposing mutations for idiopathic diseases from only one or two families, if other prior information can help trim down candidate genes into specific linkage regions or chromosomes (such as the X-linked disease Ogden syndrome [5]).

The current study was initiated by the discovery of a potential novel syndrome at The Children's Hospital of Philadelphia in 2009. We were presented with a brother and sister with a previously uncharacterized condition, which was initially suspected to be a glycogen storage disease type IV. Both children had progressive muscular weakness (myopathy) and cardiomyopathy, with normal intelligence (Table 1), and the parents were phenotypically normal. The neuromuscular weakness in both probands started around the age of 8 years, together with progressive cardiomyopathy. Additional assessment by the clinical genetics lab, with comprehensive evaluation of all known glycogen storage disease genes, failed to substantiate the hypothesis that this is a known glycogen storage disease. Given the failure of candidate gene analysis, it seemed intuitive that whole-genome techniques might be required to identify the disease-predisposing mutations.

**Table 1:** Clinical features of the syndrome, based on the three probands in two families

Feature	Description
Growth	Normal growth
Development	Normal early milestones and intelligence, presenting with neuromuscular weakness in childhood
Facial	No abnormalities noticed
Musculoskeletal	No bone deformities; progressive myopathy
Integument	Normal
Cardiac	Progressive cardiomyopathy^a^
Liver	Normal
Neurologic	Muscular weakness and muscle atrophy
Genital	Normal

^a^The severity of progressive cardiomyopathy differs between patients.

## Materials and methods

### Sample collection and characterization

**Figure 1:**
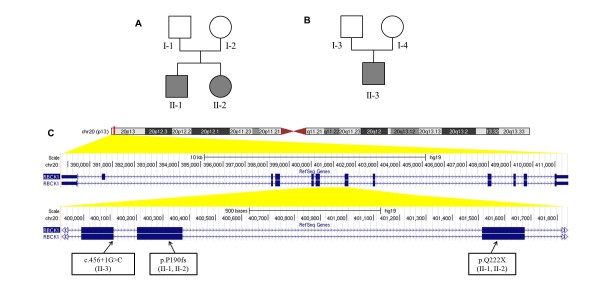
**The loss-of-function mutations within *RBCK1 *in two families**. **(a,b) **Pedigree structure for the two families, respectively. **(c) **Genome browser shots illustrating the location of the mutations within *RBCK1*. Multiple zooming levels are shown from the chromosome to the gene structure, and then to three exons harboring the mutations.

In 2009, the two affected siblings and their parents were recruited to participate in a study to identify disease genes (Figure 1a). The probands were initially suspected to have a glycogen storage disease type IV, but extensive clinical genetics testing failed to identify the disease causing gene. In late 2011, we enrolled one additional patient with comparable phenotype (Figure 1b), through a referral by the first family under study. This patient was also originally suspected to have a glycogen storage disease type IV, but all clinical genetics tests failed to identify the exact genetic cause. The Institutional Review Board of the Children's Hospital of Philadelphia has approved the study, and written informed consents were obtained from all study participants. The study conformed to the Helsinki Declaration and we have been given permission to publish the manuscript.

### SNP genotyping and data analysis

All genome-wide SNP genotyping for the family was performed using the Illumina HumanHap550 BeadChip at the Center for Applied Genomics at the Children's Hospital of Philadelphia. Standard data normalization procedures and canonical genotype clustering files provided by Illumina were used to process the genotyping signals and generate genotype calls.

The Illumina GenomeStudio software was used to process genotyping data and visualize signal intensity patterns at large-scale copy number variants (CNVs) and region-of-homozygosity (ROH) events. The log R ratio and B allele frequency measures for all markers for all samples were directly calculated and exported from the Illumina BeadStudio software. The CNV calls and ROH calls were generated using PennCNV (version 2009Aug27) [6], which utilizes an integrated hidden Markov model that incorporates multiple sources of information, including total signal intensity and allelic intensity ratio at each SNP marker, the distance between neighboring SNPs, and the allele frequency of SNPs. Family information was not used in CNV calling. The default program parameters, library files and the genomic wave adjustment routine [7] in the detect_cnv.pl program were used in generating CNV calls. The scan_region.pl program in PennCNV was used to map called CNVs to specific genes and exons, using the RefSeq gene definitions.

### Whole-genome and whole-exome sequencing

The whole-genome sequencing was performed by Complete Genomics (Mountain View, California, USA), and we provided 10 μg DNA samples to the company for the sequencing service. The DNA was sequenced with a nanoarray-based short-read sequencing-by-ligation technology [8], including an adaptation of the pairwise end-sequencing strategy [9]. The original sequence data were mapped to National Center for Biotechnology Information (NCBI) reference genome build 36 in 2010. Recently, the short reads alignment and variant calling were re-performed by the Complete Genomics pipeline version 2.2 as previously reported [8], using NCBI reference genome build 37. Each variant was assigned a quality score, which was calculated as -10 × log_10_[P(call is true)/P(call is false)], representing the confidence in the call. We removed variants that do not pass the default quality filter, including homozygous calls with quality scores less than 20, or heterozygous calls with quality scores less than 40. The variants passing the quality control threshold were used for downstream analysis.

The whole-exome sequencing was performed in house at the University of Pennsylvania. We used the Agilent SureSelect Human All Exon kit for exon capture on 5 μg input DNA samples, and then used the Illumina Genome Analyzer II platform for next-generation sequencing. We generated 137 million paired-end reads, using two separate lanes from the Genome Analyzer. Data analysis was performed using the SeqMule pipeline [10], which is an automated pipeline for analysis of high-throughput sequencing data. It integrates BWA [11], Bowtie [12], Bowtie2 [13], SOAP2 [14], SOAPsnp [15], GATK [16], SAMtools [17], VarScan [18], Picard and other popular analysis tools, and therefore gives users the flexibility to choose their preferred aligner and variant caller. In our analysis, we used the variant calls generated by the BWA alignments and GATK indel realignment procedure, similar to as previously reported [19].

### Validation by Sanger sequencing

Selected putative variants were examined among all family members using Sanger sequencing methods. Given the position of variants, the PCR primers were designed to encompass the candidate position, ensuring that common SNPs are not covered by the primers. The ABI Prism 3500 sequencer was used for sequencing, and the resulting *.AB1 files were loaded into the ABI Sequence Scanner version 1.0 for further analysis and genotype calling. All sequence traces were manually reviewed to ensure the reliability of the genotype calls.

### Variant annotation and prioritization

We used the ANNOVAR software [20] for variant annotation, analysis and filtering. Besides gene-based annotation, we used a custom 'variants reduction' pipeline to identify a list of candidate genes with the following criteria: (1) identify variants causing splicing or protein-coding changes, including stop loss and stop gain variants; (2) remove variants in the 1000 Genomes Project April 2012 release, the NHLBI-6500 Exomes (European Americans or African Americans), the CG46 database compiled from unrelated individuals sequenced by the Complete Genomics platform and the dbSNP nonFlagged database with version 137; (3) requires a recessive mode of inheritance, with at least two deleterious mutations found in each proband.

### RNA-Seq analysis

We generated Epstein-Barr virus-transformed lymphoblastoid cells for all study participants, using their peripheral blood mononuclear cells. Total RNA was extracted from cultured cells, and we made sequencing libraries using the Illumina TruSeq protocol. The Illumina HiSeq2000 sequencer was used for generating paired-end sequence data with 101 bp read length. We used the Tophat [21] version 2.0.4 and Cufflinks [22] version 2.0.2 software tools for sequence alignments and for quantifying gene expression levels. The resulting BAM files were visualized in the Integrative Genomics Viewer [23] to identify aberrant splicing patterns.

## Results and discussion

### Discovery of *RBCK1 *as a disease candidate gene

SNP genotyping revealed multiple CNVs in the two probands in the first family (Figure 1a). However, we did not identify any *de novo *CNVs or homozygous deletions shared by the two siblings. Therefore, in 2010, we started to use next-generation sequencing to comprehensively assay the genome of the patients, motivated by the successful identification of disease-predisposing genes for Mendelian diseases such as Miller syndrome and Kabuki syndrome [24,25] published in the same year.

At the time of the study, whole-genome sequencing was prohibitively expensive, and the relative merits of whole-genome versus whole-exome sequencing were not well established. Therefore, we decided to proceed with a modified approach, by sequencing one patient with whole-genome sequencing and the other by whole-exome sequencing. The whole-genome sequencing was performed by Complete Genomics, and an average fold coverage of 81× was achieved genome-wide with excellent evenness, ensuring high quality genotype calls for the patient. We identified 3,910,156 genetic variants for subsequent functional annotation and prioritization. In parallel, we performed exome sequencing in-house on the other patient. We generated 137 million paired-end reads, achieving an average coverage of 118× over designed capture regions, and with >90% of target regions covered by ≥10 reads. Therefore, the whole-genome and whole-exome data have excellent coverage statistics, even by today's standards.

We first analyzed the whole-genome data to find potential disease-predisposing genes, assuming a recessive disease model as the most likely possibility, given that this is a brother and sister pair arising from phenotypically normal parents. We utilized the ANNOVAR 'variants reduction' pipeline [20] to identify a set of candidate genes that are more likely to be the disease predisposing genes for the disease (Figure S1 in Additional file 1). Our goal is to identify a list of prioritized rare variants, and then assess the variant transmission patterns across the pedigree. We focused on the list of non-synonymous SNVs, splice variants and indels in exonic regions, given that they might be more interpretable and perhaps more likely to be disease predisposing. This pipeline leads to a list of 30 most probably disease-predisposing genes. We manually reviewed the results to remove pseudogenes and questionable variant calls due to mis-alignments (for example, *KCNJ12*, *HYDIN*), olfactory receptors (for example, *OR9G9*, *OR9G1*), as well as 'dispensable' genes with high frequency loss-of-function mutations in populations [20,26], and we were left with a list of six candidate genes. We next performed Sanger sequencing to validate these variants and examine their familial transmission patterns. We failed to validate the mutations in *LRP5 *and *MUC6*. Additionally, while we were able to validate the mutations in *FAM81B *and *MTRNR2L1*, the mutations do not follow expected inheritance patterns (both mutations come from the same parent). Therefore, two genes were left as our final set of candidate genes, including TATA box-binding protein-associated factor 1-like (*TAF1L*; MIM 607798) and RanBP-type and C3HC4-type zinc finger containing 1 (*RBCK1*; MIM 610924).

*TAF1L *is a single-exon gene that evidently arose by retrotransposition of a processed TAF(II)250 mRNA during primate evolution [27]. TAF1L is homologous to TAF(II)250 and is expressed specifically in the testis. It may act as a functional substitute for TAF1/TAF(II)250 during male meiosis, when sex chromosomes are transcriptionally silenced [27]. The two TAF1L mutations (P1266R and K1094E) are confirmed in both cases as compound heterozygotes, with each mutation inherited from a separate parent.

**Figure 2:**
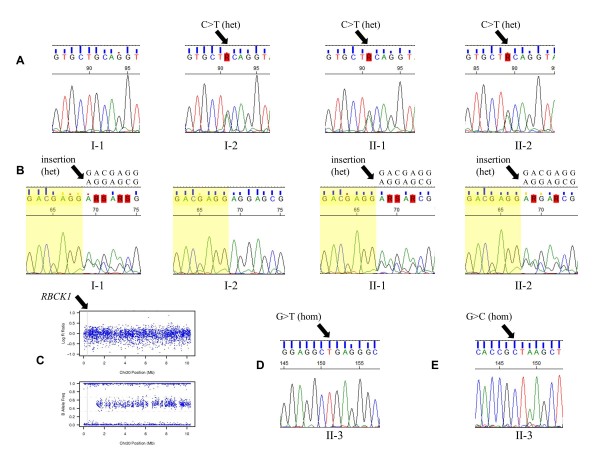
**Validation of mutations in *RBCK1 *in two families**. **(a,b) **Validation on the nonsense mutation and frameshift indel in family 1. The identified mutations are labeled with arrows. The nucleotide sequences of the insertion were resolved from the sequence trace. **(c) **SNP array showed a 1.2 Mb copy-neutral region-of-homozygosity (ROH) on chromosome 20p in the proband from family 2. The upper panel (log R ratio) represents normalized total signal intensity, demonstrating the lack of copy number changes in the 1.2 Mb region. The lower panel (B allele frequency) represents normalized allelic intensity ratio, demonstrating the lack of heterozygous SNPs in the 1.2 Mb region. The location of *RBCK1 *is marked by the grey vertical line. **(d,e) **Validation of a homozygous intronic variant and a homozygous splice variant in the proband from family 2.

*RBCK1 *is an E3 ubiquitin-protein ligase that has a pivotal role in determining the specificity of the system by recognizing target substrates [28]. *RBCK1 *is a more promising disease candidate gene, as it has two truncating mutations each inherited from one parent. These include a nonsense mutation in exon6 (p.Q222X) and a 7 bp frameshift insertion in exon5 (p.E190fs) (Figure 1c, Figure 2a, b). We note that loss of function variants in *RBCK1 *appear to be very rare in the general population; for example, there were none in the approximately 6,500 individuals in the Exome Variant Server. We also note that several other ubiquitin-protein ligase genes (*PARK2*, *NEDD4*, *TRIM9*, *NHLRC1*, *CBL*, *TRIM63*, *RNF19*, and so on) have been implicated in multiple Mendelian diseases sharing similar phenotypic features as the current patients.

We used a comparable set of procedures to analyze the exome-sequencing data. Of note, there are minimal amounts of overlap of candidate genes from whole-genome data and whole-exome data, and all the overlapping genes have been ruled out as potential candidate genes previously. We next examined why *RBCK1 *did not confirm as a candidate gene in the whole-exome data. The genome-wide variance of the heterozygous allele frequencies [29] was 0.91%, which did not suggest high amplification artifacts. Evaluation of coverage statistics confirmed the overall good coverage statistics over designed target regions in the exome (Figure S2 in Additional file 1). Additionally, on average, exons within *RBCK1 *were also well covered (Figure S3A in Additional file 1). However, the two positions with known mutations were only covered by 4 and 2 reads, respectively, and only one read contained a mutation (Figure S3B,C in Additional file 1). To further investigate this, we examined GC content around the two mutation sites, since it is known that GC content of the fragment being sequenced affects sequencing coverage [30]. Based on alignment files, the average insert fragment sizes for exome sequencing and genome sequencing were 123 bp and 358 bp, respectively. The GC content around the two mutation sites were 73.2% and 71.5%, respectively, suggesting potential issues in amplifying these fragments for exome sequencing. Finally, we also analyzed six additional exome samples sequenced on the same batch (each sample on one separate lane), and found that their coverage ranged from 0 to 6 and 1 to 2 for the two mutation sites, respectively, suggesting that poor coverage on the two mutations was a common problem for all samples. Similar challenges in exome data analysis have already been discussed before: for example, uneven coverage of exome data may result in true disease-predisposing genes being filtered out during the variant detection procedure [31]. Therefore, our results represented another example where although candidate mutations were located in the coding part of the genome, they were not detected by exon capture and sequencing.

### Validation of *RBCK1 *in a second family

As this appeared to be an extremely rare disease, we were cautious not to conclude that these mutations were definitive predisposing events for a novel syndrome at that time. In late 2011, we obtained one additional patient with comparable phenotype, through a referral by the first family under study. This patient was also originally suspected to have a glycogen storage disease type IV, but all clinical genetics tests failed to identify the exact genetic cause. Therefore, we set out to sequence all exons in *RBCK1 *using Sanger sequencing in this patient (subject II-3 in Figure 1b), though parental DNA samples were unfortunately not available for our study. Our sequencing results identified two homozygous mutations, including an intronic mutation (rs11698154) that has previously been observed in the 1000 Genomes Project [32] with a minor allele frequency of 12% (Figure 2d), as well as a previously unreported mutation (c.456+1G>C) that is located at an exon-intron boundary, apparently disrupting a canonical splicing donor site for exon 5 (Figure 2e).

Given the fact that a private mutation was called as homozygote, we suspected that this might be an artifact of variant calling, or that there is instead an exonic deletion at this position. To investigate this, we genotyped the patient using Illumina HumanHap550 SNP arrays and analyzed the signal intensity data to find deletions [6]. However, we did not observe any exonic deletions, but rather discovered that *RBCK1 *is enclosed in a 1.2 Mb copy-neutral ROH covering the p-terminal of chromosome 20 (Figure 2c). The family history did not have any evidence of consanguineous marriage, so it is likely that the *RBCK1 *ROH was due to a relatively distant shared ancestry of the two parents, or due to local uniparental isodisomy.

### Confirmation of aberrant splicing by RNA-Seq

To further validate the presence of mutations and/or their potential impacts on transcript splicing, we subsequently made Epstein-Barr virus-transformed lymphoblastoid cell lines from the patients and unaffected mother from family 1, as well as the patient from family 2. In early 2012, using RNA extracted from these cell lines, we performed transcriptome sequencing (RNA-Seq) using Illumina HiSeq2000. On average, we generated 61 million 101 bp paired-end reads for each subject.

**Figure 3:**
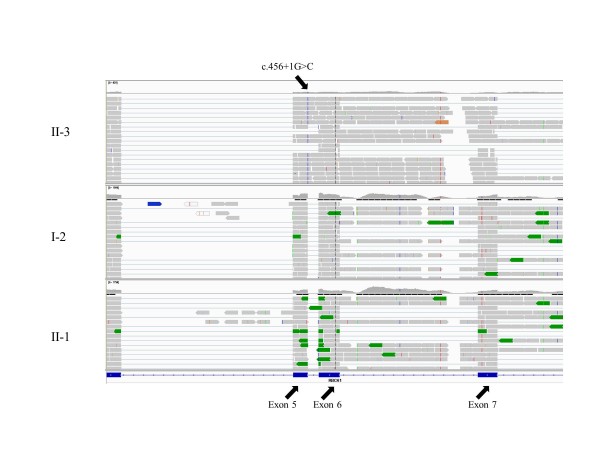
**Integrative Genomics Viewer screen shots of the RNA-Seq data on three subjects from two families**. The RNA-Seq experiments were performed using RNAs extracted from lymphoblastoid cell lines. The results validated the presence of a splice variant at the exon-intron boundary in the proband from family 2 (subject II-3), and that the intronic regions are transcribed between exon 5 and exon 6. However, the variant is not present in family 1, and exons 5 and 6 are correctly spliced in two subjects (subjects II-1 and I-2) in family 1.

For the patient in the second family (subject II-3), RNA-Seq data convincingly demonstrated the presence of a homozygous mutation at the splicing donor site in exon 5 of the *RBCK1 *gene, originally found by Sanger sequencing (Figure 3). This mutation appeared to generate a 'read-through' transcript, that is, intronic regions between exon 5 and exon 6 were also transcribed. In comparison, for the proband and his unaffected mother in the first family (subjects II-1 and I-2), RNA-Seq data showed the lack of 'read-through' transcription between exon 5 and exon 6 (Figure 3). We bioinformatically predicted the protein product for the read-through transcripts, and identified multiple pre-mature stop codons in the hypothetical protein sequence, suggesting that the transcripts may not be functional, or may be subject to nonsense-mediated mRNA decay. Interestingly, we also noted that all three subjects had intronic transcription between exon 6 and exon 7. However, examination of RNA-Seq data sets in other projects suggested that these intronic transcripts exist in all other RNA-Seq samples, so they may be due to the presence of unannotated exons or other non-coding RNAs.

The gene function for *RBCK1 *was not well characterized, but it was reported to be a component of E3 ubiquitin-protein ligase, which accepts ubiquitin from specific E2 ubiquitin-conjugating enzymes, such as UBE2L3/UBCM4, and then transfers it to substrates [28]. Recently, a study related *HOIL1 *(*RBCK1*) deficiency to a fatal human disorder with immunodeficiency, autoinflammation and amylopectinosis [33]. The authors demonstrated that NF-κb activation in response to IL-1β was compromised in patients' fibroblasts, but the patients' mononuclear leukocytes, particularly monocytes, were hyper-responsive to IL-1β. However, we note that the authors did not prove that the variants were causal for the observed phenotypes, since the fibroblast cells from patients may have harbored other variants. We were unable to garner any evidence on immunodeficiency or auto-inflammation in the three probands from two families in our study, although they both have clear signs of amylopectinosis (glycogen storage disease type IV), which was the very reason they were referred to us. We also cannot exclude the possibility that different mutations in the same *RBCK1 *gene may lead to distinct and unrelated phenotypes (immune-related problems and amylopectinosis). Despite the lack of direct functional evidence associating the mutations with the amylopectinosis phenotype, the discovery of a genetic cause further establishes that this phenotype of interest may represent a novel syndrome.

In conclusion, whole-genome sequencing identified a mutation in *RBCK1 *as possibly predisposing to a novel, extremely rare Mendelian disease. Together with several recently published studies [1-3], this example illustrates the possibility to identify disease-predisposing mutations for novel idiopathic diseases using a very limited number of patient samples. However, we also caution that extensive functional validations are required to assess why loss of function in the candidate gene leads to the observed disease phenotypes. Finally, our study also represents an example where exome sequencing failed to identify disease genes due to lack of comprehensive coverage and/or even coverage of the target regions. With the ever-decreasing cost of whole-genome sequencing, we expect that whole-genome sequencing will be used much more in the near future for finding the genetic causes of Mendelian disorders.

## Abbreviations

bp: base pair; CNV: copy number variant; IL: interleukin; NCBI: National Center for Biotechnology Information; PCR: polymerase chain reaction; ROH: region of homozygosity; SNP: single nucleotide polymorphism.

## Competing interests

The study was partly supported by a donation from the first family described in the article.

## Authors' contributions

KW: RG, YG, JB and GL analyzed the data and advised on data analysis. FGO and CH generated PCR and Sanger sequencing results. KT and CK generated the SNP genotyping results. ET helped with phenotype assessment. KW drafted the manuscript, and all authors reviewed and edited the manuscript. KW and HH designed the study and HH supervised all aspects of the study. All authors read and approved the final manuscript.

## Supplementary Material

Additional File 1 **Figures S1, S2 and S3**. Click here for file
