# Supplementary material for: Whole-genome DNA/RNA sequencing identifies truncating mutations in RBCK1 in a novel Mendelian disease with neuromuscular and cardiac involvement
Source: Genome Med. 2013 Jul 26;5(7):67. doi: 10.1186/gm471 (PMC3971341; doi:10.1186/gm471)

Supplementary Figure 1. Illustration of the variants reduction procedure on two probands in the first family. One proband was sequenced by whole-genome sequencing, and another proband was sequenced by whole-exome sequencing. Applying a recessive model of disease inheritance, we identified 30 and 4 candidate genes in the two probands, without any shared gene.


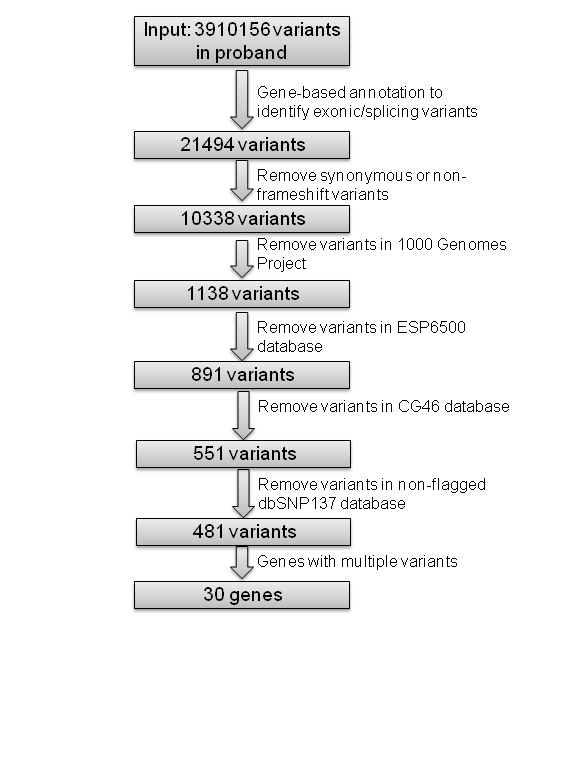

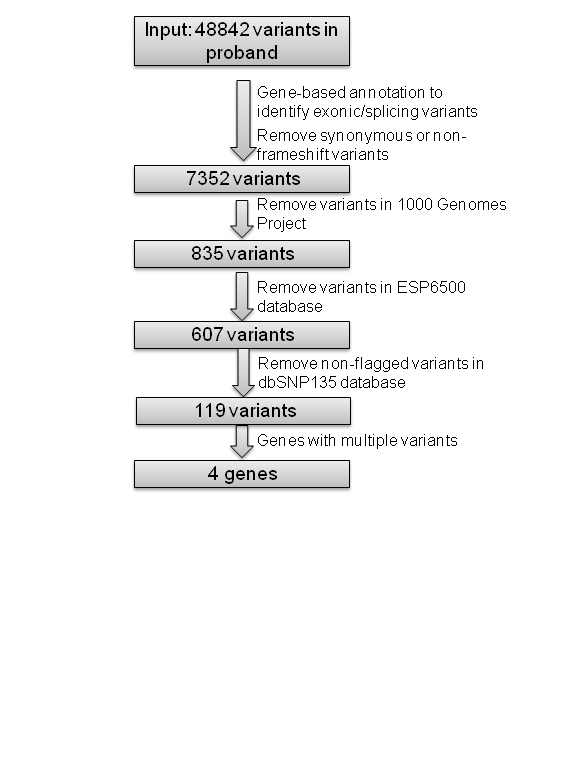


Supplementary Figure 2. Distribution of coverage over target regions in the exome sequencing data on subject II-2. We generated 137 million paired-end reads, achieving an average coverage of 118X over designed capture regions. Over 90% of target regions were covered by ≥10 reads, suggesting overall good quality of the exome sequencing data.


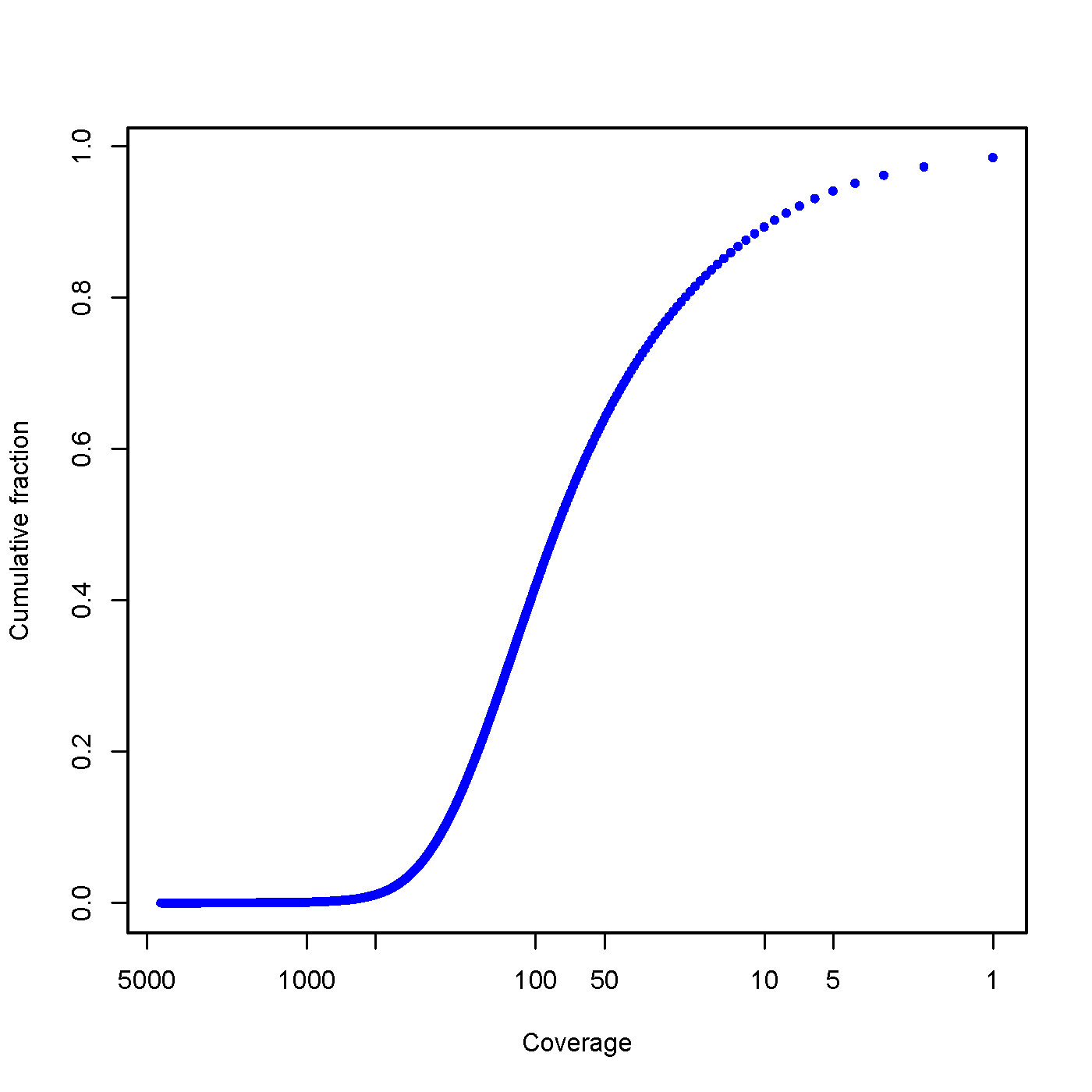


Supplementary Figure 3. Integrative genomics viewer screen shot of *RBCK1* in whole-exome sequencing data. (A) coverage of the entire gene (B) coverage plot on the region around p.P190fs, where the candidate position is marked by two vertical lines (C) coverage plot on the region around p.Q222X, where the candidate position is marked by two vertical lines. Although the gene was generally covered well in exome sequencing data, the two sites were covered by only four and two reads, respectively, and a mutation is detected in only one read.


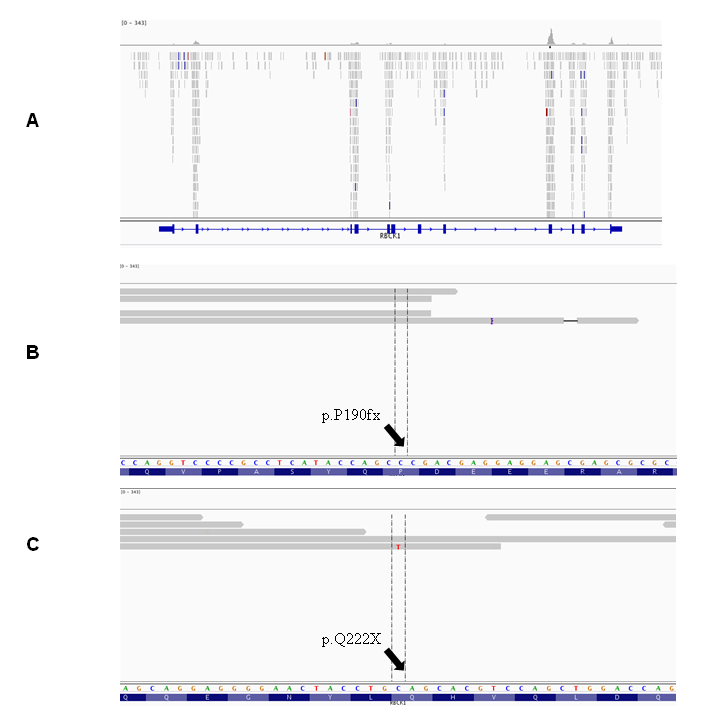

Supplement: Additional File 1 — Figures S1, S2 and S3. [file gm471-S1.DOCX]
